# The impact of sports participation on subjective wellbeing among Chinese young adults: exploring the chain mediation effect of perceived social support and psychological resilience

**DOI:** 10.3389/fpubh.2026.1841181

**Published:** 2026-05-13

**Authors:** Xinchun Wang, Minmin Du, Tongnian Yang, Zhihui Li, Huiyu Shi, Jian Yang, Xinrou Wan

**Affiliations:** 1 College of Physical Education and Health, East China Normal University, Shanghai, China; 2 School of Sports Science, Qufu Normal University, Jining, China; 3 School of Athletic Performance, Shanghai University of Sport, Shanghai, China; 4 Kunming Xishan Hubin Middle School, Kunming, China

**Keywords:** perceived social support, psychological resilience, sports participation, subjective wellbeing, young adults

## Abstract

**Introduction:**

This study aims to examine the association of sports participation with young adults' subjective well-being and to explore its underlying pathways by analyzing the mediating and chain mediating effects of perceived social support and psychological resilience between the two variables.

**Methods:**

Using data from the 2021 Chinese General Social Survey (CGSS), statistical analyses including variance analysis, correlation analysis, regression analysis, and mediation analysis were conducted using SPSS 27.0 and its Process V4.3 component.

**Results:**

The results indicate that sports participation is positively associated with subjective well-being among young adults. Perceived social support and psychological resilience independently mediate this relationship, while also forming a significant chain mediation pathway. Among these pathways, perceived social support exerts the most significant mediating effect.

**Conclusion:**

These findings reveal the importance of both social and psychological resources in explaining how sports participation is associated with subjective well-being, provide theoretical foundations and practical insights for further exploring key factors influencing subjective well-being, and effectively promote the physical and mental health development of young adults.

## 1 Introduction

Ensuring and realizing a happy life for the people has always been one of the highest goals of governance for the Communist Party of China and the Chinese government. In March 2025, the Government Work Report of the 14th National People's Congress proposed to “safeguard and improve people's livelihoods, delivering tangible gains in their sense of fulfillment, happiness, and security” (1). However, the United Nations' 2024 World Happiness Report ranked China 60th among 145 surveyed countries, below Japan and South Korea (2). As a core component of social structure and a key agent of action, the subjective wellbeing of young adults directly impacts social participation effectiveness and sustainable development capacity. Against the backdrop of contemporary social transformation and the national strategies of building a sports powerhouse and a healthy China, how to enhance young adults' subjective wellbeing through sports participation has become a central research topic in academia.

Current research on subjective wellbeing primarily focuses on physiological, psychological, and medical perspectives. Freedman employed a dual psychological and medical approach to investigate subjective wellbeing among older adults with disabilities (3), while Shi assessed the impact of mitochondrial genes on individual wellbeing using UK Biobank data (4). Research subjects have predominantly been disease patients and older populations. Naber examined the extent to which atypical antipsychotics improve patients' subjective wellbeing (5), while Nguyen's study emphasized the importance of culturally relevant strategies for enhancing quality of life across diverse aging populations (6).

Among the various factors influencing subjective wellbeing, sports participation, perceived social support, and psychological resilience serve as intervention tools for physical activity and emotional wellbeing, playing a positive role in enhancing young adults' subjective wellbeing. Individuals who engage in sports report higher levels of life satisfaction, and the frequency of participation is a key determinant. Only regular engagement in physical activities produces positive effects (7). Perceived social support is recognized as one of the most effective resources for alleviating individual life and work stress, significantly enhancing subjective wellbeing (8), with particularly pronounced effects among young adults (9). Psychological resilience levels are closely linked to young adults' subjective wellbeing. Research indicates that family environments shape psychological resilience by fostering effective communication, cultivating optimistic attitudes toward adversity, providing robust support, and developing problem-solving skills—all of which enhance the capacity to perceive happiness (10). According to social cognitive theory and the stress buffering model (11, 12), perceived social support and psychological resilience exhibit a dynamic interactive relationship: the former provides the social resource foundation for the latter, while the latter enhances individuals' effective utilization of social support. Together, they constitute the psychosocial mechanism for enhancing subjective wellbeing. In the context of sports participation, individuals receive social feedback through interactions with peers and groups, thereby strengthening their perceived social support. Meanwhile, the challenges and stress inherent in sports activities allow social support to play a critical role in buffering stress and facilitating emotion regulation, thus providing essential conditions for the development of psychological resilience. This finding suggests that the influence of sports participation on the subjective wellbeing of young adults is unlikely to operate solely through a direct pathway and is instead mediated by the combined effects of social relational resources and individual psychological resources. Focusing only on the direct association between sports participation and subjective wellbeing may therefore be insufficient to fully capture the underlying psychosocial mechanisms through which this influence occurs. In addition, previous research has emphasized the influence of sociodemographic factors on subjective wellbeing. For example, health status and economic income status constitute the physiological and material foundations of individuals' quality of life and serve as important determinants of subjective wellbeing (13). Meanwhile, demographic characteristics such as gender and age exhibit significant heterogeneity in individuals' perceptions of stress and experiences of wellbeing (14).

Existing research, both domestic and international, has generated substantial findings on the relationship between sports participation and subjective wellbeing, but considerable scope for further development remains. Previous research has rarely incorporated perceived social support and psychological resilience within the same analytical model for simultaneous examination. This limitation is particularly evident in studies focusing on young adults, where systematic and integrative analyses of the underlying mechanisms through which sports participation influences subjective wellbeing remain limited. Building on this, drawing on relevant research frameworks (15), this study incorporates perceived social support and psychological resilience into a unified research framework examining the relationship between sports participation and the subjective wellbeing of young adults. A chain mediation model is then constructed to further investigate the influencing factors and underlying mechanisms of subjective wellbeing in this population, thereby providing theoretical insights into how their wellbeing is shaped.

## 2 Literature review and research hypotheses

### 2.1 Sports participation and subjective wellbeing

In previous studies, sports participation referred to individuals personally engaging in physical activities primarily involving bodily movement (i.e., physical participation in exercise) (16). However, with the ongoing development of modern theories in sports sociology, sport has gradually transcended the narrow scope of purely physical activity and evolved into a comprehensive socio-cultural lifestyle. From the perspective of multidimensional involvement theory, individuals' sports participation is not limited to physical participation but also involves substantial cognitive and emotional involvement (17). Accordingly, the present study conceptualizes sports participation as individuals' participation in sport-related activities through either physical or psychological involvement, with the aim of promoting physical and mental health, facilitating social interaction, and enriching emotional experiences. This includes not only physical forms of participation, such as participating in physical exercise, but also mental forms of participation, such as watching sports events.

Subjective wellbeing is an individual's overall assessment and subjective experience of life quality based on self-defined standards (18). Professor Martin E.P. Seligman, founder of positive psychology, categorizes happiness into three types: pleasure-based, engagement-based, and meaning-based. Engagement-based happiness refers to the joy derived from wholehearted immersion in activities (19). Thus, young adult sports participation serves as a primary pathway to achieving engagement-based happiness, manifesting in both physical and psychological health dimensions. Physically, sports participation stimulates the release of neurotransmitters like endorphins, promoting relaxation and subjective wellbeing (20). Regarding psychological wellbeing, participating in physical activity and watching sports events enables young adults to experience pleasurable, positive psychological states (21). This enhances self-confidence, life satisfaction, and health benefits, thereby increasing subjective wellbeing (22). Furthermore, research indicates that sports participation significantly influences subjective wellbeing, with the two exhibiting a positive correlation (23). Based on this, it is proposed that:

H1: Sports participation positively influences young adults' subjective wellbeing.

### 2.2 Mediating role of perceived social support

Perceived social support refers to an individual's subjective feelings of being respected, understood, and cared for by their social network. Compared to objective social support, this subjective support encompasses a broader scope, enhancing an individual's adaptive capacity when facing stress and alleviating negative emotions (24). As posited by the main effect and buffering effect models, social support plays a crucial role in individual mental health development (25). It can mitigate the negative impact of adverse external environments, improve quality of life, and maintain higher levels of wellbeing (26). Physical activities provide participants with avenues for interpersonal interaction and help build extensive social networks (27). Regular participation in physical exercise can effectively enhance perceived social support, thereby increasing subjective wellbeing (28). Furthermore, research indicates that physical exercise significantly influences perceived social support, with the two exhibiting a positive correlation (29). Separately, it is well established that perceived social support positively promotes subjective wellbeing (30). Based on this, it is proposed that:

H2a: Sports participation positively influences young adults' perceived social support.
H2b: Sports participation promotes young adults' subjective wellbeing through the mediating effect of perceived social support.

### 2.3 Mediating role of psychological resilience

Psychological resilience refers to an individual's capacity for adaptation and recovery when facing stress, setbacks, or major life changes. This ability not only helps individuals effectively cope with challenges but also enables them to grow after adversity, achieving a better psychological state than before (31). In social environments, individuals must adapt to stressors or adversity, prompting continuous learning and adjustment. This process enhances stress resilience while mobilizing resources to overcome challenges, thereby strengthening psychological resilience (32). Resilient individuals maintain emotional stability during difficult situations, leading to more positive evaluations of their quality of life and sustained positive affective experiences (33). The collaborative and competitive nature of sports activities provides young adults with favorable conditions to enhance psychological resilience. Sports participation can improve adverse physical and mental states, reduce stress responses (34), and thereby induce changes in an individual's psychological resilience (35). Simultaneously, psychological resilience exhibits a strong positive predictive effect on subjective wellbeing (36). Based on this, it is proposed that:

H3a: Sports participation positively influences psychological resilience among young adults.
H3b: Sports participation promotes subjective wellbeing among young adults through the mediating effect of psychological resilience.

### 2.4 The chain mediation effect of perceived social support and psychological resilience

Perceived social support and psychological resilience are closely interrelated. Social support serves as an external protective factor for psychological resilience, positively promoting resilience in young adults and effectively buffering psychological stress caused by adverse life events (37). Research indicates that high levels of perceived social support enhance stroke patients' capacity and resources for actively coping with adversity, elevating their psychological resilience and thereby alleviating post-stroke depressive symptoms (38). Social support theory posits that individuals with broader social support networks demonstrate stronger coping abilities in challenging situations (39), which further enhances psychological resilience (40). Participating in physical activity promotes interpersonal interaction (41), enabling individuals to obtain effective perceived social support. This gradually builds and strengthens their belief in social capital, equipping them with the capacity to cope with and adapt to adversity, thereby enhancing psychological resilience (42) and ultimately increasing subjective wellbeing. Based on this, it is proposed that:

H4a: Perceived social support positively influences psychological resilience among young adults.
H4b: Sports participation promotes young adults' subjective wellbeing through the chain-mediated effects of perceived social support and psychological resilience.

**Figure 1:**
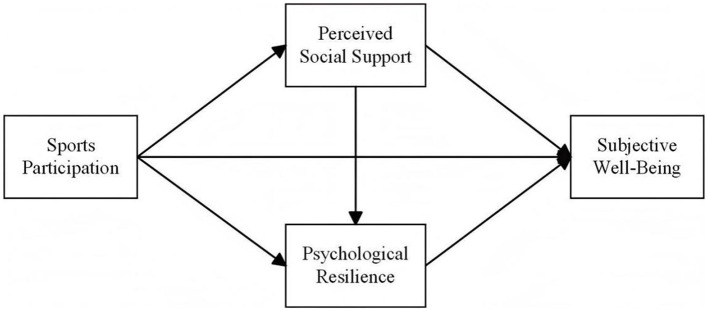
Hypothetical model.

The hypothetical model of this study is illustrated in Figure 1.

## 3 Research design and methods

### 3.1 Data sources

This study utilizes data from the 2021 Chinese General Social Survey (CGSS). The CGSS is one of the earliest national, comprehensive, and continuous academic survey programs in China. It aims to systematically collect multilevel data on society, communities, families, and individuals in order to identify trends in social change and examine issues of major scientific and societal significance. The target population consists of adult residents aged 18 years and above in mainland China. The survey sample covers all provinces, municipalities, and autonomous regions across the country, making CGSS one of the most widely used and authoritative data sources for research on Chinese society.

The World Health Organization (WHO) has referred to young adults as individuals aged 18–44 years (43). However, in sociological research contexts, the age range of young adults may exhibit a degree of contextual flexibility across different socio-cultural settings. Within the current stage of social development and role structure in China, individuals under the age of 45 generally remain in an active phase of career development and family responsibility. This stage is typically characterized by relatively stable physical functioning and sustained patterns of sports participation. Therefore, based on the WHO age reference and in consideration of the research focus and sample characteristics of the present study, the target population was defined as young adults aged 19–45 years.

In the data preprocessing stage, this study strictly followed objective criteria for sample screening to ensure data quality and minimize potential selection bias. First, the original dataset was restricted to respondents aged 19–45 years, and individuals outside this age range were excluded to ensure that the sample corresponded to the target population of young adults. Second, some variables used in this study were derived from specific thematic subsample modules of the CGSS database. Due to the questionnaire routing and split-sample design employed in the survey, certain items inevitably contained completely random missing values. To avoid potential bias introduced by statistical imputation, a listwise deletion approach was adopted, whereby all cases with missing values on any of the core variables were removed. In addition, to reduce the potential influence of careless or inattentive responses, a rigorous data quality control procedure was implemented. Specifically, invalid responses characterized by patterned answering, substantial logical inconsistencies across items, or extreme outliers were carefully identified and removed. After these procedures, a total of 685 high-quality valid samples were retained for the final analysis.

### 3.2 Variable description

The dependent variable in this study is subjective wellbeing. Following relevant research (44), it was measured using the item: “Overall, do you feel your life is happy?” This question was measured using a seven-point Likert scale. During the data processing stage, reverse scoring was applied, with values ranging from 1 (“Completely unhappy”) to 7 (“Completely happy”). Higher scores indicate greater subjective wellbeing.

The independent variable in this study is sports participation. From a psychosocial perspective, particularly within the contemporary socio-cultural context of China, both active physical exercise and passive viewing of sporting events represent important pathways through which young adults engage with the sport domain. Following relevant research (45), data were measured using two items: “In the past year, how often did you engage in the following activities during your free time? (Participating in physical exercise, watching sports events).” Each question offered five response options, with values ranging from 1 (Never) to 5 (Daily). Considering that this variable consisted of only two items, the Spearman–Brown split-half reliability coefficient was used to assess its reliability. The results indicated that the Spearman–Brown coefficient for the two items was 0.72, suggesting good internal consistency. In this study, the mean of the two items was calculated to construct a continuous indicator of sports participation, with higher scores indicating more frequent participation.

The mediating variables in this study were perceived social support and psychological resilience. Perceived social support refers to individuals' subjective perception of emotional connection, communication quality, and a sense of social belonging derived from their social networks—particularly family members and peer groups (46). Unlike objectively received tangible assistance, this construct emphasizes individuals' subjective evaluations of the adequacy of their social networks and the quality of interpersonal interactions. To assess this construct, three items from the questionnaire were selected to capture its multidimensional characteristics: “I feel that most people seem to have more friends than I do,” “I feel particularly happy when I am with my family,” and “I sometimes find it difficult to communicate with my family.” All items were rated on a six-point Likert scale, ranging from 1 (“Completely disagree”) to 6 (“Completely agree”). Among these, “I feel that most people seem to have more friends than I do” and “I sometimes find it difficult to communicate with my family” were negatively worded and were therefore reverse-coded during data processing (i.e., 1 = 6, 2 = 5, …, 6 = 1) to ensure that higher scores consistently reflected higher levels of perceived social support. After confirming internal consistency (Cronbach's α = 0.71), the mean score of the three items was calculated to construct a continuous variable ranging from 1 to 6, with higher scores indicating higher levels of perceived social support.

Referencing related research (47), psychological resilience is conceptualized not merely as a passive defense against adversity but, more centrally, as an individual's capacity for experiential transformation, goal orientation, and cognitive maturation developed through life challenges. Data were collected using three items: “As I grow older, I have learned many lessons from life that have made me stronger and more capable.” “Most of the life goals I set motivate rather than discourage me,” and “I feel pleased that my perspectives have become increasingly mature over the years.” All items were rated on a six-point Likert scale, ranging from 1 (“Completely disagree”) to 6 (“Completely agree”). This measurement module demonstrated good internal consistency (Cronbach's α = 0.75). In this study, the mean score of the three positively worded items was calculated to form a continuous variable ranging from 1 to 6, with higher scores indicating greater psychological resilience. (see Table 1).

**Table 1:** Variable settings and description.

Type	Name	Variable declaration
Dependent variable	Subjective wellbeing	Overall, do you feel your life is happy? Please rate on a scale of 1–7, where completely unhappy = 1, very unhappy = 2, somewhat unhappy = 3, neutral = 4, somewhat happy = 5, very happy = 6, and completely happy = 7.
Independent variable	Sports participation	In the past year, how often did you engage in the following activities during your free time? (Participating in physical exercise, watching sports events) Please rate your frequency on a scale of 1–5: never = 1, several times a year or less = 2, aeveral times a month = 3, several times a week = 4, and daily = 5.
Mediating variables	Perceived social support	To what extent do you agree with the following statements: “I feel that most people seem to have more friends than I do,” “I feel particularly happy when I am with my family,” and “I sometimes find it difficult to communicate with my family.” Please indicate your response on a six-point Likert scale: completely disagree = 1, disagree = 2, slightly disagree = 3, slightly agree = 4, agree = 5, and completely agree = 6.
	Psychological resilience	To what extent do you agree with the following statements: “As I grow older, I have learned many lessons from life that have made me stronger and more capable.” “Most of the life goals I set motivate rather than discourage me,” and “I feel pleased that my perspectives have become increasingly mature over the years.” Please indicate your response on a six-point Likert scale: completely disagree = 1, disagree = 2, slightly disagree = 3, slightly agree = 4, agree = 5, and completely agree = 6.
Control variables	Gender	Male = 1, female = 2.
	Age	Survey year minus birth year.
	Household registration	Agricultural household registration = 1, non-agricultural household registration = 2.
	Health status	Very unhealthy = 1, somewhat unhealthy = 2, average = 3, somewhat healthy = 4, and very healthy = 5.
	Economic income status	How would you rate your family's economic income relative to the average level in your local area? Please indicate your response on a five-point scale: far below average = 1, below average = 2, average = 3, above average = 4, and far above average = 5.

To further verify the construct validity of the combined items, a confirmatory factor analysis was conducted on the six items constituting the mediating variables. The results indicated that the two-factor model met all statistical fit criteria (χ^2^/*df* = 2.85, CFI = 0.965, TLI = 0.921, RMSEA = 0.052, SRMR = 0.038), and its fit was significantly better than that of a single-factor competing model combining all items (Δχ^2^(1) = 28.45, *p* < 0.001), demonstrating satisfactory structural validity.

To avoid estimation biases caused by other variables influencing young adults' subjective wellbeing, this study incorporates a series of individual-level and household-level control variables based on relevant literature (48, 49). These specifically include the following variables: gender, age, household registration, health status, and economic income status. These variables were selected based on existing research findings indicating their potential influence on young adults' subjective wellbeing. They were thus incorporated as control variables in the analysis to more accurately estimate the effects of the independent and mediating variables. Descriptive statistics for all variables are presented in Table 2.

**Table 2:** Descriptive statistics of variables.

Variable name	Value range/Category	Mean/Frequency	Standard deviation/Percentage	Skewness	Kurtosis
Sports participation	1–5	2.239	0.751	0.231	0.050
Perceived social support	1–6	4.251	0.768	−0.352	−0.059
Psychological resilience	1–6	4.806	0.531	−1.150	3.003
Subjective wellbeing	1–7	5.073	0.805	−0.712	4.201
Age	19–45	31.542	7.493	—	—
Health status	1–5	3.952	0.882	—	—
Economic income status	1–5	2.709	0.688	—	—
Gender	Male	309	45.109%	—	—
	Female	376	54.891%	—	—
Household registration	Agricultural household registration	457	66.715%	—	—
	Non-agricultural household registration	228	33.285%	—	—

### 3.3 Data processing

This study employed SPSS 27.0 for statistical analysis. First, the normality assumption for all continuous variables was assessed. The results (see Table 2) showed that the absolute skewness values of the core variables ranged from 0.231 to 1.150, all below 2, and the absolute kurtosis values ranged from 0.050 to 4.201, all below 7, indicating that the data approximately met the assumption of a normal distribution (50). Therefore, the use of Pearson correlation analysis was appropriate and robust. Second, independent-samples *t*-tests and one-way ANOVAs were conducted to examine differences in core variable scores across demographic characteristics, and Pearson correlation analysis was employed to assess relationships among variables. Finally, controlling for covariates, mediation effects were tested using Model 6 in the SPSS PROCESS component, with 5,000 bootstrap samples used to estimate 95% confidence intervals for each path, thereby evaluating the significance of the mediation effects.

## 4 Research findings

### 4.1 Common method bias examination

As all CGSS questionnaire data were collected through self-reporting by respondents, common method bias could potentially exist. Therefore, this study conducted a Harman single-factor test on the questionnaire data. Using SPSS 27.0, an unrotated exploratory factor analysis was performed on sports participation, perceived social support, psychological resilience, and subjective wellbeing. The results showed two factors with eigenvalues greater than 1. The first factor explained 28.27% of the variance, below the 40% critical threshold, suggesting that common method bias is unlikely to be a serious concern in this study.

### 4.2 Analysis of differences in research variables

To verify the appropriateness of the selected control variables, independent-samples *t*-tests and one-way ANOVAs were conducted to examine differences in the core variables across demographic and socioeconomic characteristics. The results (see Table 3) indicated that, for the independent variable sports participation, young male adults reported significantly higher participation levels than young female adults (*t* = 1.962, *p* < 0.05). Further analysis indicates that the effect size of this difference (*d* = 0.147) is small, suggesting that its practical significance is limited. In addition, young adults with non-agricultural household registration reported significantly higher participation levels than those with agricultural household registration (*t* = −2.591, *p* < 0.01). Regarding the dependent variable subjective wellbeing, both health status (*F* = 12.545, *p* < 0.001) and economic income status (*F* = 9.523, *p* < 0.001) showed highly significant main effects. In addition, health status was also significantly associated with differences in perceived social support (*F* = 10.793, *p* < 0.001) and psychological resilience (*F* = 3.400, *p* < 0.01). Moreover, economic income status exerted a highly significant effect on perceived social support (*F* = 8.914, *p* < 0.001).

**Table 3:** Comparison of differences among variables.

Variable	Category	Sports participation	Perceived social support	Psychological resilience	Subjective wellbeing
Gender	Male	2.301 ± 0.732	4.302 ± 0.718	4.804 ± 0.541	5.063 ± 0.740
	Female	2.190 ± 0.771	4.209 ± 0.811	4.808 ± 0.522	5.069 ± 0.862
	*T*	1.962^*^	1.572	−0.105	−0.116
Household registration	Agricultural household registration	2.191 ± 0.792	4.226 ± 0.803	4.807 ± 0.534	5.040 ± 0.852
	Non-agricultural household registration	2.340 ± 0.673	4.289 ± 0.711	4.802 ± 0.543	5.120 ± 0.701
	*T*	−2.591^**^	−1.041	0.111	−1.218
Health status	—	—	—	—	—
	*F*	8.687^***^	10.793^***^	3.400^**^	12.545^***^
Economic income status	—	—	—	—	—
	*F*	6.641^***^	8.914^***^	1.073	9.523^***^

^*^p < 0.05, ^**^p < 0.01, ^***^p < 0.001.

### 4.3 Correlation analysis of research variables

**Figure 2:**
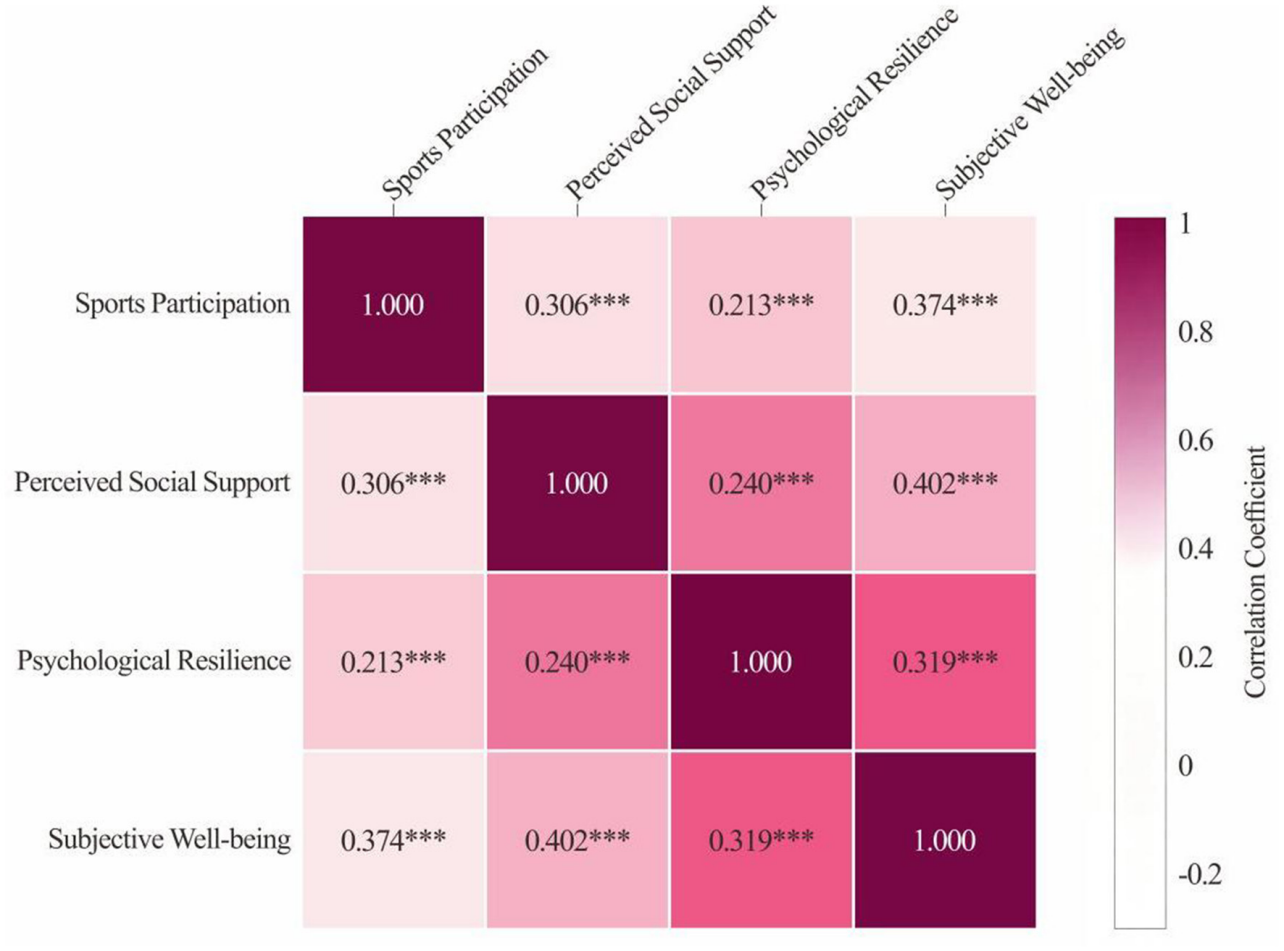
Correlation analysis of variables. *p < 0.05, **p < 0.01, ***p < 0.001.

The correlation analysis results indicate (see Figure 2) that sports participation is significantly positively correlated with perceived social support, psychological resilience, and subjective wellbeing scores (*p* < 0.001). Subjective wellbeing is significantly positively correlated with perceived social support and psychological resilience scores (*p* < 0.001). Perceived social support is significantly positively correlated with psychological resilience scores (*p* < 0.001).

### 4.4 Chain mediation effect analysis

Before conducting the chain mediation and regression analyses, this study assessed the regression model for multicollinearity and the assumptions related to the error terms. First, the results of the multicollinearity test indicated that the variance inflation factors for the independent variable, mediating variables, and control variables ranged from 1.022 to 1.209, all below 10. Meanwhile, the tolerance values ranged from 0.827 to 0.979, all above 0.1, indicating that no multicollinearity was present among these variables. Second, regarding the assumptions of the error terms, the Durbin–Watson statistic for testing the independence of residuals was 1.588, which falls within the acceptable range of 1.5 to 2.5. This result suggests that the residuals were independent and that no serious first-order autocorrelation was present. In addition, the skewness and kurtosis of the standardized residuals were −0.619 and 3.478, respectively, satisfying the assumption of approximate normality under large-sample conditions.

Based on this, the present study employed Model 6 of the SPSS PROCESS component to examine the chain mediation model, controlling for gender, age, household registration, health status, and economic income status. The Bootstrap method was applied with 5,000 samples to estimate the 95% confidence intervals for each effect (51). The mediation effect was examined with sports participation as the independent variable, perceived social support and psychological resilience as mediating variables, and subjective wellbeing as the dependent variable. Regression analysis revealed (see Table 4):

(1) After controlling for gender, age, household registration, health status, and economic income status, sports participation positively influenced subjective wellbeing, with a significant direct effect (*B* = 0.231, *p* < 0.001), supporting Hypothesis H1.
(2) Sports participation significantly and positively influenced perceived social support (*B* = 0.264, *p* < 0.001). Perceived social support significantly and positively influenced subjective wellbeing (*B* = 0.266, *p* < 0.001). The confidence interval for the mediating effect of perceived social support was [0.042, 0.105], which did not include zero. Hypotheses H2a and H2b were supported, with indirect mediation accounting for 20.349% of the total effect.
(3) Sports participation significantly and positively influenced psychological resilience (*B* = 0.111, *p* < 0.001), and psychological resilience significantly positively influenced subjective wellbeing (*B* = 0.297, *p* < 0.001). The confidence interval for the mediation effect of psychological resilience was [0.014, 0.057], which did not include zero. Hypotheses H3a and H3b were supported, with indirect mediation accounting for 9.593% of the total effect.
(4) Perceived social support significantly and positively influenced psychological resilience (*B* = 0.122, *p* < 0.001). The confidence interval for the chained mediating effect of perceived social support on psychological resilience was [0.004, 0.018], which did not include zero. Hypotheses H4a and H4b were supported by the data, with the chained mediating effect accounting for 2.907% of the total effect.

**Table 4:** Regression analysis of chain mediation model.

Variable	Perceived social support		Psychological resilience		Subjective wellbeing	
	*B*	*T*	*B*	*T*	*B*	*T*
Gender	−0.055	−0.991	0.037	0.929	0.070	1.313
Age	0.012	3.231^**^	0.004	1.592	−0.003	−0.827
Household registration	0.015	0.247	−0.018	−0.421	0.016	0.279
Health status	0.157	4.832^***^	0.045	1.913	0.093	2.907^**^
Economic income status	0.127	3.076^**^	−0.012	−0.411	0.091	2.280^*^
Sports participation	0.264	6.971^***^	0.111	3.993^***^	0.231	6.075^***^
Perceived social support	–	–	0.122	4.456^***^	0.266	7.115^***^
Psychological resilience	–	–	–	–	0.297	5.727^***^
*R* ^2^	0.149		0.089		0.289	
*F*	19.737^***^		9.409^***^		34.297^***^	

^*^p < 0.05, ^**^p < 0.01, ^***^p < 0.001.

**Figure 3:**
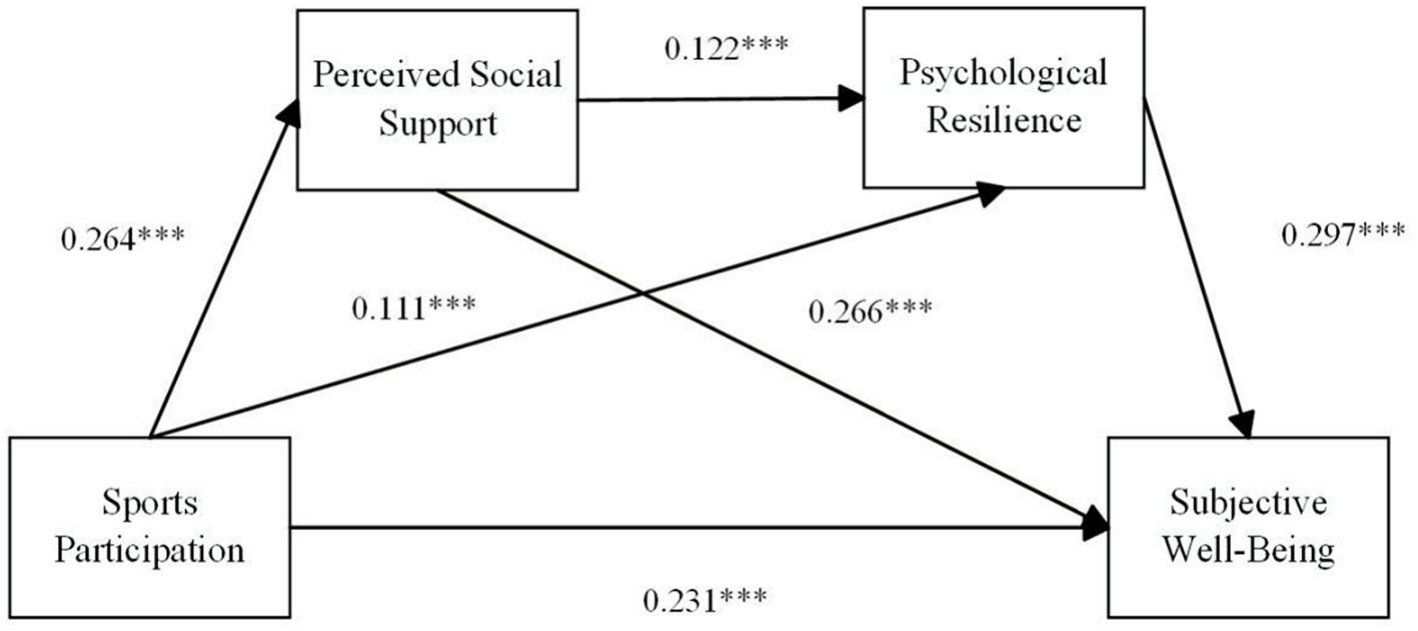
Chain mediation model. *p < 0.05, **p < 0.01, ***p < 0.001.

Mediation analysis revealed (see Table 5, Figure 3) that perceived social support and psychological resilience significantly mediated the relationship between sports participation and subjective wellbeing. The total unstandardized mediation effect size was 0.113, accounting for 32.849% of the total effect. The mediating effect comprises three specific pathways: sports participation → Perceived social support → Subjective wellbeing (effect size: 0.070); Sports participation → Psychological resilience → Subjective wellbeing (effect size: 0.033); Sports participation → Perceived social support → Psychological resilience → Subjective wellbeing (effect size: 0.010).

**Table 5:** Mediation effect analysis.

Path	Effect	Proportion (%)	Boot SE	Bootstrap 95%CI	
				Lower limit	Upper limit
Total effect	0.344	100	0.039	0.268	0.420
Direct effect	0.231	67.151	0.038	0.156	0.306
Total indirect effect	0.113	32.849	0.020	0.077	0.154
Sports participation → Perceived social support → Subjective wellbeing	0.070	20.349	0.016	0.042	0.105
Sports participation → Psychological resilience → Subjective wellbeing	0.033	9.593	0.011	0.014	0.057
Sports participation → Perceived social support → Psychological resilience → Subjective wellbeing	0.010	2.907	0.004	0.004	0.018

## 5 Discussion and analysis

### 5.1 Direct effect of sports participation on young adults' subjective wellbeing

A key finding of this study is that sports participation is not merely a statistically significant correlate but also constitutes an important pathway for enhancing the subjective wellbeing of young adults, thereby supporting Hypothesis H1. The observed direct effect (*B* = 0.231, *p* < 0.001) further underscores the substantive role of regular participation in physical activity in shaping individuals' overall life evaluations. This finding extends previous research (52) by demonstrating that the positive effects of sports participation are not limited to short-term emotional improvements but may also contribute to more stable and enduring perceptions of wellbeing.

Self-determination theory posits (53) that intrinsic motivation stems from humans' innate exploratory tendencies, serving as the internal driving force for sports participation. Higher levels of intrinsic motivation among young adults make them more likely to perceive physical exercise as a social activity contributing to a fulfilling life. The three fundamental psychological needs of autonomy, competence, and relatedness constitute the core motivational mechanisms underlying sports participation. When young adults experience autonomy in choosing activity formats, perceive self-efficacy through skill improvement, and establish social connections via interaction during exercise, their intrinsic motivation system enters a positive activation state, effectively enhancing subjective wellbeing.

From the perspective of theoretical elaboration, the promotive effect of sports participation on subjective wellbeing arises not only from the immediate satisfaction of basic psychological needs but also from its role in facilitating the internalization of individual motivation over time. During engagement in or watching sports events as positive sport experiences, motivations that initially stem from external regulation or health-related pressures are gradually integrated into the self. As a result, sports participation shifts from an externally driven requirement to an internally endorsed choice. This process of motivational internalization enhances psychological coherence and self-integration, enabling wellbeing to develop from a transient emotional response into a relatively stable psychological state. Compared with short-term effects driven by external incentives, the sustained activation of intrinsic motivation, together with its internalization, offers a more convincing explanation for the long-term beneficial effect of sports participation on subjective wellbeing.

A consensus has emerged in the field of exercise and sports psychology: participating in physical exercise significantly promotes individual wellbeing. Research indicates (54) that young adults with high levels of intrinsic motivation are more likely to view physical activity as an extension of self-actualization rather than an external constraint. This manifests as individuals progressively integrating socially advocated fitness values into personally embraced practices. Such internal motivation generates more enduring persistence and positive emotional experiences compared to external reward and punishment mechanisms.

Overall, from the perspectives of motivational development and psychological integration, this study elucidates the underlying mechanisms through which sports participation influences subjective wellbeing and provides theoretical support for understanding its long-term beneficial effects.

### 5.2 Independent mediating role of perceived social support

Perceived social support, as an independent mediating variable, positively influences young adults' subjective wellbeing, and this mediating effect was significant, supporting hypotheses H2a and H2b. This finding aligns with prior empirical studies (55).

The independent mediating effect of perceived social support accounted for the largest proportion (20.35%), indicating that, in the process through which sports participation influences young adults‘ subjective wellbeing, obtaining external social support plays the most central and critical role. Within the social context of sports, participants can effectively expand and strengthen their social networks through physical interactions with opponents and communicative exchanges with peers or family members. This dynamic interpersonal process provides individuals with diverse support, effectively releases negative emotions, and significantly enhances their social skills and emotional management abilities. For young adults, the positive social networks built through sports activities offer robust support for the development of their social-emotional competencies, thereby forming a virtuous cycle mechanism of “social interaction—capacity enhancement—emotional development.” The accumulation of social capital gained through sports participation significantly promotes young adults' subjective wellbeing (56).

Sports participation offers young adults a vital platform for socialization, expanding their social networks and providing material, spiritual, and emotional assistance and support. This effectively alleviates the psychological burdens and emotional distress experienced by this unique demographic amid modern society's fast-paced lifestyle and high achievement expectations. This virtuous cycle ultimately translates into the effective accumulation of psychological resources, fostering an increase in subjective wellbeing. The findings of this study further extend the “stress-buffering hypothesis” in the contemporary context. Drawing on interdisciplinary research in sociology and psychology (57), young adults today, who experience pervasive digital socialization but limited high-quality in-person connections, face elevated risks of latent loneliness and social isolation. Sports participation provides a high-quality, real-world social environment grounded in “shared goals” and “embodied co-presence.” The perceived social support arising from this environment is not only more authentic and trustworthy than virtual interactions but also effectively buffers against psychological exhaustion induced by modern work and life stressors, serving as a critical social resource that sustains subjective wellbeing among young adults.

### 5.3 Independent mediating role of psychological resilience

Psychological resilience, as an independent mediating variable, had a positive effect on young adults' subjective wellbeing, with an effect size of 0.033. This mediating effect was significant, supporting hypotheses H3a and H3b, and is broadly consistent with previous research (58, 59).

Psychological resilience represents an internalized form of psychological capital that, once developed, remains relatively stable and provides a continuous internal buffer, helping to maintain baseline levels of subjective wellbeing. In contemporary society, characterized by high-intensity competition and a fast-paced lifestyle, young adults occupy a critical life stage, transitioning from education to career, forming close relationships, and assuming family and social responsibilities, inevitably encountering multiple real-world pressures and novel challenges. Young adults with high levels of psychological resilience tend to adopt proactive coping strategies when faced with life setbacks or work-related stress, perceiving adversity as an opportunity for personal growth rather than as an insurmountable threat. Furthermore, young adults have largely completed the exploration of self-identity and are beginning to assume adult social roles and responsibilities. The autonomous problem-solving abilities, independent decision-making skills, and self-confidence encompassed by psychological resilience align closely with young adults' intrinsic developmental needs to pursue independence and prove their self-worth. Therefore, within the mediating pathway of sports participation influencing subjective wellbeing, psychological resilience plays a more fundamental, stable, and developmentally relevant role for young adults.

According to psychological resilience theory (60), physical exercise can increase psychological capital and cultivate resilience, enhancing the ability to withstand external risks. When participating in physical activity, young adults often face multiple challenges, including difficulties in skill acquisition, competitive pressures, and negative emotional experiences resulting from losses. In the short term, these factors may adversely affect young adults' psychological state. However, in the long run, through persistent personal effort and with support from peers and family, young adults who successfully overcome setbacks not only develop stronger adaptability and resilience but also enhance their capacity to address future life and work challenges. A deeper exploration of the underlying mechanisms, drawing on the Cross-Stressor Adaptation Hypothesis (61), helps explain this process from two perspectives. On the one hand, the physiological stress experienced during moderate physical exercise can induce adaptive changes in the sympathetic nervous system and the hypothalamic pituitary adrenal (HPA) axis. On the other hand, watching sports events as a form of psychological engagement not only provides vicarious cognitive resources for coping with frustration through athletes' displays of perseverance but also evokes high-arousal emotional responses through the dynamic fluctuations of competition, thereby creating a safe context in which individuals can practice emotional regulation. More importantly, the stress adaptation mechanisms and emotion regulation capacities developed through participating in physical activity and watching sports events can be transferred across situations and effectively applied when young adults cope with challenges in their daily work and life. Therefore, sports participation is not only a means of enhancing physical fitness but also serves as a form of repeated psychological stress training that ultimately strengthens the psychological resilience of young adults.

It is important to further note that the explanatory power of the psychological resilience model in the present study (*R*^2^ = 0.089) is relatively modest, which, to some extent, reflects the inherent complexity and multidimensional nature of psychological resilience as a construct. As a dynamically evolving psychological resource, psychological resilience may be associated with behavioral factors and social resources and may also be shaped by individuals' internal characteristics and developmental environments. From a theoretical perspective, the development of psychological resilience involves interactions between individual-level traits, including personality characteristics and emotion regulation capacities, and external contextual factors such as family support systems and early-life stress experiences. Therefore, the variables included in this study primarily capture the roles of behavioral factors and social resources in shaping psychological resilience, rather than encompassing its broader range of determinants. These findings further suggest that understanding how sports participation influences psychological resilience requires situating this relationship within a more comprehensive psychological and socio-ecological framework, thereby enabling a more holistic understanding of the mechanisms underlying its development.

### 5.4 The chain mediation effect of perceived social support and psychological resilience

This study reveals that perceived social support and psychological resilience exert a chain mediation effect on the influence of sports participation on young adults' subjective wellbeing. The mediation effect is significant, confirming research hypotheses H4a and H4b. The underlying mechanism is that perceived social support gained through sports participation builds psychological resources for young adults, including emotional support, cognitive guidance, and instrumental assistance. These resources are internalized through social interaction and gradually develop into the stable psychological trait of psychological resilience, enabling young adults to build the capacity to buffer stress and cope effectively with adversity (*B* = 0.122, *p* < 0.001). The enhanced psychological resilience then exerts a more stable and direct promotion on subjective wellbeing by facilitating positive emotion regulation, enhancing problem-solving efficacy, and maintaining an optimistic orientation (*B* = 0.297, *p* < 0.001). This internalization pathway, characterized by the process of external support leading to internal resilience and subsequently to wellbeing experience, reveals that sports participation not only influences wellbeing through immediate social interactions but also provides young adults with a stable internal foundation for happiness by cultivating resilient psychological qualities through this mediating mechanism.

To gain a deeper understanding of this chain mechanism, the conservation of resources theory (62) can be adopted as an explanatory framework, positing that individuals strive to acquire, retain, and protect the resources they value. In the initial stage of sports participation, young adults first acquire external conditional resources, exemplified by perceived social support. Through continued exercise engagement within a supportive environment, these external resources effectively reduce individuals' anxiety over potential resource loss when facing challenges, gradually internalizing into personal trait resources, namely psychological resilience. This evolution of resources, from the development of external social networks to the strengthening of internal psychological defense mechanisms, ultimately fosters a sustained and profound experience of subjective wellbeing among young adults.

Furthermore, the internalization process of the aforementioned resources should be understood within the broader socio-contextual realities faced by Chinese young adults. At present, young adults are confronted with multiple challenges, including intensified academic competition, increasing employment uncertainty, heightened family expectations, and rising housing pressures. Under the cumulative impact of these stressors, individuals are more likely to experience sustained psychological burden and emotional depletion, thereby placing greater demands on psychological resilience. Within this context, perceived social support functions not only as an external resource but also plays a critical role in buffering the effects of stress through emotional bonding and social interaction. Particularly in the context of sports participation, individuals may continuously strengthen their sense of social connectedness and group identity through peer interaction, team cooperation, and shared goal orientation. This process facilitates the transformation of support resources embedded in social relationships into stable capacities for psychological regulation and adaptation. Against this dynamic backdrop of contextual stress, social support, and resource internalization, sports participation provides an important practical pathway for the development of psychological resilience, thereby further reinforcing its indirect contribution to subjective wellbeing.

In addition, this study integrates physical activities and watching sports events into a broader construct of sports participation. Although this approach allows for a more comprehensive representation of young adults' overall sports participation, it may also, to a certain extent, obscure differences in the underlying psychosocial mechanisms associated with these two forms of participation. Physical activities are more likely to foster psychological resilience through embodied practice, skill-related challenges, and emotional regulation processes, whereas watching sports events may primarily enhance perceived social support through social identification, emotional resonance, and interaction in shared viewing contexts. Therefore, the mediating pathways identified in this study are more appropriately interpreted as reflecting the overall psychosocial effects of sports participation as a general construct, rather than indicating equivalence in the mechanisms underlying these two distinct forms of engagement.

However, it should be noted that the effect size of the chain mediation pathway identified in this study is 0.010, accounting for only 2.907% of the total effect, indicating a relatively limited overall contribution. This finding suggests that, in the relationship between sports participation and subjective wellbeing, this pathway is more likely to function as a refined psychological transmission mechanism rather than a dominant one. To some extent, this result reflects the nature of subjective wellbeing as a comprehensive psychological outcome characterized by multiple developmental pathways, which typically arise from the interaction of diverse psychological and social factors. Accordingly, the influence transmitted through the sequential mechanism of perceived social support and psychological resilience is more likely to manifest as a gradual strengthening of individuals' psychological adaptation rather than a strong effect driven by a single pathway. In this sense, the chain mediation effect identified in the present study helps to further elucidate how sports participation indirectly influences subjective wellbeing through the interplay between social relational resources and individual psychological resources. This finding provides process-oriented evidence for understanding the psychological effects of sports participation and offers a valuable complement to future research aimed at advancing the exploration of underlying mechanisms from an integrated multi-pathway perspective. On this basis, it is also necessary to adopt a cautious stance regarding the scope of interpretation of the aforementioned pathways. Because this study is based on cross-sectional data, the findings primarily reflect associations among variables rather than causal relationships. Therefore, the pathway through which sports participation influences subjective wellbeing through perceived social support and psychological resilience should be interpreted as a theoretically plausible explanatory framework rather than a definitive causal conclusion. Future research should employ longitudinal designs or experimental methods to further examine the causal directions among these variables and their dynamic mechanisms.

Compared to existing single-mediation models, this study reveals a chain-like mediation pathway linking perceived social support and psychological resilience. The mediation effect is interpreted as follows: sports participation enhances young adults' perceived social support levels, which in turn promotes the development of psychological resilience, ultimately improving wellbeing indices through this resilience. This not only aligns with social psychology's perspective on the reciprocal influence of emotions and behavior but also clearly delineates the dynamic process of transforming perceived external social resources into internal psychological adaptability. It offers a more refined, developmentally grounded integrative perspective for analyzing the underlying mechanisms through which sports participation enhances young adults' subjective wellbeing.

This study quantifies the contributions of different pathways, demonstrating that the core mechanism through which sports participation influences subjective wellbeing lies in emotional regulation rather than mere behavioral substitution. It necessitates a dual approach of social construction and emotional support. If young adults experience negative emotions and remain unsupported over the long term, the subsequent effects may evolve into adverse symptoms, potentially harming individuals, families, and society. Therefore, perceived social support and psychological resilience serve not only as an endogenous safeguard for young adults to gain wellbeing through sports participation but also as a vital resource for young adults to continuously realize their potential and pursue happiness.

Moreover, the covariates controlled in this study also warrant attention. Importantly, while sociodemographic factors such as better health status and higher economic income status provide important support for subjective wellbeing, the psychosocial benefits associated with sports participation, particularly the enhancement of perceived social support and psychological resilience, remain highly robust after controlling for these covariates. This finding highlights that sports participation, as an efficient and broadly applicable intervention strategy, can effectively enhance the subjective wellbeing of young adults across different socioeconomic backgrounds. In contrast, although the gender difference in sports participation reached statistical significance, the effect size was relatively small, and the significance level was close to the threshold. This suggests that the observed gender difference reflects a relatively modest group difference rather than a core determinant with strong explanatory power. In large-sample studies, even small effects may reach statistical significance. Therefore, the interpretation of such findings should take effect size into account to avoid overemphasizing statistical significance and to enable a more cautious evaluation of their practical significance and explanatory value.

### 5.5 Study limitations

The present study has several limitations. First, young adults' subjective wellbeing was assessed using a single-item self-report. Although suitable for large-scale surveys, this approach does not capture the multidimensional structure of wellbeing; thus, the findings reflect overall levels rather than specific dimensions. Future research should adopt multidimensional measures for more nuanced assessment. Second, the composite measure of sports participation may involve behavioral heterogeneity. While both physical exercise and watching sports events are associated with higher subjective wellbeing, their underlying psychosocial mechanisms may differ. Future studies should model these behaviors separately to compare their distinct pathways and better capture the multidimensional nature of sports participation. Third, the measurement of mediating variables was constrained by the original questionnaire design. Compared with established psychological scales, these items are limited in content coverage and construct completeness. Future research should employ more systematic multidimensional scales to better capture the complex structures of perceived social support and psychological resilience. Fourth, this study is based on a survey of Chinese young adults. While the measures are suitable for macro-level sociological and psychological assessments, they are not intended for individual clinical diagnosis. The generalizability of the findings to professional athletes, other age groups, and different cultural contexts should therefore be interpreted with caution. Future research should include cross-cultural samples, broader age ranges, and specific subpopulations to further enhance external validity. Fifth, this study treats Chinese young adults aged 19–45 as a single analytical group, despite the potential developmental heterogeneity within this broad age range. Differences in social support structures, psychological resilience, and pathways to subjective wellbeing may exist across age subgroups. Future research should adopt more fine-grained age-stratified designs to examine potential variations in mediation pathways across developmental stages, thereby providing more developmentally sensitive and targeted insights. Sixth, although Harman's single-factor test suggested that common method bias is unlikely to be serious, it may not adequately detect such bias. Therefore, potential bias cannot be entirely excluded, and future studies should employ more rigorous approaches, such as CFA-based methods.

## 6 Conclusions and implications

### 6.1 Conclusions

This study examined the direct and indirect association between sport participation and subjective wellbeing among young adults through the chain mediation role of perceived social support and psychological resilience. The findings indicate that sport participation not only directly predicts subjective wellbeing but also exerts indirect effects through perceived social support, psychological resilience, and their sequential mediation. Overall, the present study underscores the importance of social and psychological resources in explaining how sport participation contributes to wellbeing. Future research is needed to verify these relationships using longitudinal or experimental designs and to test the generalizability of the proposed mechanism across diverse populations and contexts.

### 6.2 Implications

Based on the research findings, the present study proposes the following practical implications:

(1) At the school and community levels, efforts should not be limited to providing sports facilities. Instead, priority should be given to designing sports programs that emphasize cooperation and sustained interaction, such as class-based leagues, club competitions, community team activities, and partner-based exercise programs. These initiatives can strengthen young adults' sense of social connectedness, belonging, and perceived social support during participation.
(2) At the family and social organization levels, greater attention should be paid to the positive role of watching sports events as a form of vicarious or psychological participation. Activities such as family co-viewing, campus-based group viewing, and community viewing events can transform sports events into meaningful contexts for social interaction, emotional resonance, and value identification among young adults. In addition, such engagement can be further guided to extend from interest in watching sports events to actual participation in physical activity, thereby broadening the practical pathways through which sports participation enhances subjective wellbeing.
(3) At the level of sports program design, greater emphasis should be placed on fostering psychological resilience. Grassroots sports organizations, schools, and workplaces can incorporate elements such as moderate challenges, tiered goal setting, team collaboration, and post-activity reflection into sports activities. These approaches can support young adults in progressively enhancing their emotional regulation, coping capacity in the face of adversity, and positive cognitive appraisal when dealing with exercise-related difficulties, competitive pressure, and experiences of failure.
(4) From a longer-term practical perspective, sports-based interventions aimed at enhancing young adults' subjective wellbeing should not be limited to increasing participation frequency. Instead, they should be systematically designed around a continuous pathway that integrates sports participation and watching sports events with the accumulation of social support and the strengthening of psychological resilience. Only by embedding sports participation within authentic contexts of social interaction and psychological development can its sustained effect on promoting subjective wellbeing among young adults be fully realized.

## Data Availability

The data supporting the conclusions of this article are available from the Chinese General Social Survey (CGSS). Access to the dataset requires prior user registration and approval through the official platform.
